# De Novo Assembly and Comparative Transcriptome Profiling of *Anser anser* and *Anser cygnoides* Geese Species’ Embryonic Skin Feather Follicles

**DOI:** 10.3390/genes10050351

**Published:** 2019-05-08

**Authors:** Cornelius Tlotliso Sello, Chang Liu, Yongfeng Sun, Petunia Msuthwana, Jingtao Hu, Yujian Sui, Shaokang Chen, Yuxuan Zhou, Hongtao Lu, Chenguang Xu, Yue Sun, Jing Liu, Shengyi Li, Wei Yang

**Affiliations:** 1 College of Animal Science and Technology, Jilin Agricultural University, Changchun 130118, China; sello@jlau.edu.cn (C.T.S.); xinrongjielu@163.com (C.L.); petunia.msuthwana@gmail.com (P.M.); suiyujian2000@163.com (Y.S.); joe199697@163.com (Y.Z.); luhongtao4755@163.com (H.L.); x924299@163.com (C.X.); ningxin20121216@163.com (Y.S.); L1145545376@163.com (J.L.); 18104476170@163.com (S.L.); 18443984846@163.com (W.Y.); 2 Key Laboratory for Animal Production, Product Quality and Safety of Ministry of Education, Changchun 130118, China; 3 Beijing General Station of Animal Husbandry, Beijing 100107, China; chenshaokang-21@126.com

**Keywords:** feather development, gene expression, Illumina HiSeq 4000, trinity software

## Abstract

Geese feather production and the quality of downy feathers are additional economically important traits in the geese industry. However, little information is available about the molecular mechanisms fundamental to feather formation and the quality of feathers in geese. This study conducted de novo transcriptome sequencing analysis of two related geese species using the Illumina 4000 platform to determine the genes involved in embryonic skin feather follicle development. A total of 165,564,278 for *Anser anser* and 144,595,262 for *Anser cygnoides* clean reads were generated, which were further assembled into 77,134 unigenes with an average length of 906 base pairs in *Anser anser* and 66,041 unigenes with an average length of 922 base pairs in *Anser cygnoides.* To recognize the potential regulatory roles of differentially expressed genes (DEGs) during geese embryonic skin feather follicle development, the obtained unigenes were annotated to Gene Ontology (GO), Eukaryotic Orthologous Groups (KOG), and Kyoto Encyclopedia of Genes and Genomes (KEGG) for functional analysis. In both species, GO and KOG had shown similar distribution patterns during functional annotation except for KEGG, which showed significant variation in signaling enrichment. *Anser asnser* was significantly enriched in the calcium signaling pathway, whereas *Anser cygnoides* was significantly enriched with glycerolipid metabolism. Further analysis indicated that 14,227 gene families were conserved between the species, among which a total of 20,715 specific gene families were identified. Comparative RNA-Seq data analysis may reveal inclusive knowledge to assist in the identification of genetic regulators at a molecular level to improve feather quality production in geese and other poultry species.

## 1. Introduction

Geese are an economically important agricultural poultry species raised worldwide with the ability to produce high quality meat, fatty liver, as well as feathers and down [1]. Birds are covered with feathers, which are peculiar appendages of the epidermal and dermal layers of the skin [2]. Feather development and growth occur at the bottom of a dermal papilla in the follicle. Follicular morphogenesis and the first premature feathers, named as the natal down, first begin to grow during the embryonic development period [3]. There are three major divisions of feathers in birds: downy feathers (radially symmetrical) derived from secondary feather follicles; contour feathers (bilaterally symmetrical); and flight feathers (bilaterally asymmetrical) derived from primary feather follicles [4]. Evolution in birds has resulted in feathers adopting various forms in morphology, color, and mechanical properties not only amongst different poultry species, but also among the different individual body parts, hence providing a useful model to investigate the phenotypic variations at the molecular level of related species [5]. There is well-documented detailed information on the growth, morphogenesis, and structural features of fowl feather follicles [6,7]. Even though there exist some precise similarities in the general characteristics of feather follicle formation in avian, there are still substantial variations in feather morphogenesis within and between species. The prenatal downy follicles in geese are further subdivided in primary and secondary feather follicles that have evolved autonomously of each other and form levels in a linear arrangement. In postnatal life, primary follicles with the greater diameter progress to contour and flight feathers, whereas the secondary follicles of a smaller diameter appear later and develop specifically into downy feathers [8]. The nutritional and environmental conditions “indirectly” influence the quality and quantity of feathers while genetics “directly” influence the quality and quantity of feathers [9]. Furthermore, the quantity and quality of geese downy feathers characteristics vary considerably within and between breeds [10]. Geese downy feathers and soft feathers provide high quality insulation materials for bedding and clothing, and hence serve as supreme economical traits [11]. However, there have been few discoveries about the variations in molecular mechanisms that regulate geese feather follicle development between closely related species.

Understanding the genomic and transcriptional regulators of complex functional mechanisms in closely related animal species require the universal and comparative analysis of biological and cellular processes [12]. Next generation sequencing (NGS) is an advanced technology used in large-scale transcriptome analysis and provides a novel opportunity to generate large amounts of gene expression data in a given time [13]. High-throughput RNA-sequencing (RNA-Seq) is a cost-effective NGS molecular technique, which is widely used in the mass gene expression profiling of living organisms, including nonmodel species with restricted genomic information [14]. RNA-Seq independently relies on prior information of the gene sequences of the target species, hence transcriptomic reconstruction is useful for molecular genetic researches in nonmodel species [15]. The genomic information, including gene expression analysis, Simple Sequence Repeat (SSR), gene and transcription factor (TF) discoveries, is acquired through the vital step of the de novo assembly by sequencing mass data reads [16]. *Anser cygnoides* are light-bodied geese species characterized by a higher reproduction rate, better environmental adaptability, and enhanced disease resistance capacity, which are indicators of good original material and the natural gene pool of crossbreed predominance and high performance [17]. In our previous study, we used the transcriptome analysis of geese (*Anser anser*) embryonic skin feather follicles from embryonic day 13 (E13), embryonic day 18 (E18), and embryonic day 28 (E28) to identify various gene expression profiles by using the de novo transcriptome sequencing analysis [18]. In the present study, we have expanded our research by the transcriptome sequencing of *Anser cygnoides* geese to compare the expression pattern profiling with geese (*Anser anser*) at the last day of embryonic development (E28) before hatching. A comparative transcriptome analysis will provide good knowledge of the functional molecular mechanisms associated with feather follicle formation and different gene expressions and signaling pathways, which might be helpful for future investigations as molecular markers to improve downy feather breeding in geese.

## 2. Materials and Methods

### 2.1. Ethics Statement

All the experimental procedures on animals were carried out in coherence with appropriate guidelines established by the Ministry of Agriculture of the People’s Republic of China. This study was approved by the Animal Health and Care Committee of Geese Industry Research and Development Centre of Jilin Agricultural University (Approval number: GR(J)18-006. Date: 29 March 2018; GR(J)18-008. Date: 13 April 2018).

### 2.2. Animal Sample Collection

Seventy-two eggs from each species were collected from a geese breeding research center (Jilin Agricultural University, Jilin Province, Northeast of China). The eggs were incubated in one incubator, but in separate racks following the farm daily incubation procedures. At embryonic day 28 (E28), three embryos for each species were randomly selected to conduct this study. The cervical dislocation method was applied to slaughter the sampled geese embryos to obtain the embryonic skin across the dorsal regions about 1 cm^2^ around the midline and from two wings. These were rinsed separately with water pretreated by diethylpyrocarbonate to cleanse the samples and inactivate RNases [19]. All of the samples were immediately put in liquid nitrogen and stored in a −80 °C refrigerator for RNA isolation.

### 2.3. RNA Isolation

The total RNA of the sampled dorsal skin tissues was isolated using the TRIzol Reagent (Invitrogen Life Technologies, Carlsbad, CA, USA), according to the manufacturer’s instructions. The total RNA for each sample was treated with DNAse 1 (Ambion/Life Technologies) to remove genomic DNA contamination. A Nanodrop 2000 (Thermo Fisher Scientific, Waltham, MA, USA) with the absorbance at 260 nm was used to determine the concentration and purity of the RNA. The quality of the RNA was detected by 1.2% agarose gel electrophoresis and the integrity of RNA was measured by using an Agilent 2100 Bio analyzer (Agilent, Santa Clara, CA, USA). 

### 2.4. Library Sequencing

The total RNA for each sample was used to prepare the library sequencing by (Invitrogen Life Technologies, Carlsbad, CA, USA) following the manufacture’s protocol. Briefly, poly (A)-containing mRNA was purified using oligo(dT) cellulose. The first-strand complementary DNA (cDNA) was synthesized from short mRNA fragments, which were reverse-transcribed and amplified with random primers to form double-stranded cDNA. The cDNA library was used as the template to perform Polymerase Chain Reaction (PCR) amplification for fragment enrichment and 1.2% RNase-free agarose gel electrophoresis was used to make the ligation products size fractioned. A RNase H. TruSeq Stranded RNA Sample Preparation Kit (Illumina) was used to generate sequencing libraries. An Illumina HiSeq 4000 platform (Gene Denovo Co., Guangzhou, China) was used for sequencing six cDNA libraries from each species. The raw sequencing data has been uploaded in the National Center for Biotechnology Information (NCBI) sequence read archive (SRA) under the accession numbers SRP156879 and PRJNA521094. 

### 2.5. De Novo Assembly and Functional Annotation

The open-source software FastQC (www.bioinformatics.babraham.ac.uk/projects/fastqc) was used to check and visualize the raw reads. The obtained raw data from the libraries was trimmed to remove low quality reads and adaptor sequences. Reads with more than 10% unknown nucleotide base pairs were discarded. De novo assembly of the high-quality reads from the six accessions was performed by Trinity software (version: 2.1.1, Broad Institute of Massachusetts Institute of Technology and Harvard, Cambridge, MA, USA) with default parameters and no reference sequence. The longest transcripts in the cluster units were considered as nonredundant unigenes. The functionality of the assembled transcripts was predicted in comparison with BLASTX searches in the NCBI non-redundant (Nr) protein database (available online: http://www.ncbi.nlm.nih.gov), Swiss-Prot protein database (available online: http://www.expasy.ch/sprot), KEGG pathway database (available online: http://www.genome.jp/kegg), and KOG database (available online: http://www.ncbi.nlm.nih.gov/COG) using the BLASTN algorithms cut-off (E-value < 10^−5^). The direction of the unigene sequence from the four databases was determined based on the high scoring alignments. The discrepancy between the alignment resulted from the employed databases, and the merits of the results were accredited following this order: Nr, Swiss-Prot, KEGG, and KOG. Unigene sequence mismatches in any of four databases entries, coding regions, and sequence direction were determined by using expressed sequence tags (ESTs) Scan. 

### 2.6. Differentially Expressed Genes (DEGs) Analysis

Unigene annotations provide information regarding the expression and functional annotation of the identified genes. Clean unigene read sequences were mapped into the transcriptome reference databases by using BLASTX (E-value < 10^−5^). Based on the annotations in the protein databases, Blast2GO was used to obtain GO annotations for the aligned unigene sequences, the Web Gene Ontology Annotation Plot (WEGO) software was used to establish GO functional classifications for all unigenes, and the KEGG database was used to obtain pathway annotations (E-value threshold 10^−5^). The gene expression analysis was estimated by using the RPKM (reads per kilobase per million mapped reads) method [20]. The differentially expressed genes (DEGs) were defined with a |log2 Fold Change| > 1 using a threshold of false discovery rates (FDR < 0.05) and the corresponding value threshold was statistically significant. Meanwhile, gene expression data were normalized to log_2_^(Anser anser/Anser cygnoides)^.

### 2.7. RNA-Seq Data Validation by Real Time Quantitative Polymerase Chain Reaction (RT-qPCR)

RNA-Seq data reliability was validated by conducting RT-qPCR. Eight DEGs: collagen type I alpha 1 chain (COL1A1); tumor protein, translationally-controlled 1 (TPT1); glyceraldehyde-3-phosphate dehydrogenase (GAPDH); aldehyde dehydrogenase 2 family member (ALDH2); Wnt family member 5B (WNT5B); SRP receptor beta subunit (SRPRB); transforming growth factor beta regulator 1 (TBRG1); and sorting nexin 3 (SNX3) were randomly selected. Total RNA was extracted from each species through embryonic skin feather follicle samples collected for RNA-Seq. cDNA was then synthesized using a ReverTra Ace qPCR RT Kit (TOYOBO, Osaka, Japan) from 1 μg of the same total RNA samples; the *β -actin* gene was used as the reference house-keeping gene. SYBR Green Real time PCR Master Mix (TOYOBO, Osaka, Japan) was used to perform the qPCR reactions in an Applied Biosystems 7500 Real-Time PCR System (Thermo Fisher Scientific Inc.,Waltham, MA, USA) with a 20 μL reaction system comprising 10 μL of SYBR Green Real time PCR Master Mix, 0.8 μL of each of the forward and reverse primers (200 μM), 2 μL of cDNA, and 6.4 μL of distilled water. The RT-qPCR program was 95 °C for 60 s; followed by 40 cycles of 95 °C for 15 s, 60 °C for 15 s, and 72 °C for 45 s; and ended with a final stage of melting curve analysis. The relative quantification of gene expression was detected in triplicate per sample. Relative expression values were calculated using the 2^−ΔΔCt^ method.

## 3. Results

### 3.1. Illumina cDNA Data Sequencing and De Novo Assembly

**Figure 1:**
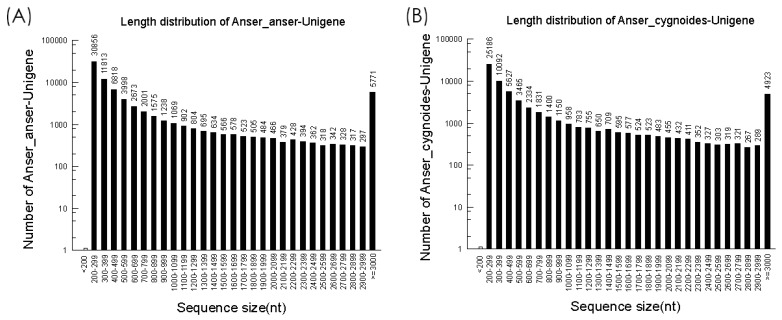
Summary of the unigene length distribution after Trinity assembly (≥200 nucleotides) for both species. The highest unigene numbers were observed from the 200–299 nucleotides range with 30,856 and 25,186 unigenes, respectively.

Three RNA-Seq were constructed from both *Anser anser* and *Anser cygnoides*. The cDNA libraries were generated and transcriptome sequencing was performed using an Illumina sequencing platform in order to obtain highly reliable de novo transcriptome assembly for each species. First, open-source software FastQC (www.bioinformatics.babraham.ac.uk/projects/fastqc) was used to evaluate the sequence information. The total of 170,246,914 raw reads for *Anser anser* were generated and 147,754,320 raw reads for *Anser cygnoides* (Table 1). After disposal of adaptor, low quality sequences, and reads from ribosomal RNA (rRNA) contaminations, the high-quality reads were de novo assembled into unique transcripts using Trinity software resulting in 165,564,278 and 144,595,262 clean reads obtained for *Anser anser* and *Anser cygnoides*, respectively. Moreover, the clean reads generated from *Anser anser* consisted of 24,315,965,489 nucleotides and *Anser cygnoides* produced 21,274,765,140 nucleotides. Furthermore, the obtained clean sequences were de novo assembled into unique transcripts (unigene). For *Anser anser*, 77,134 total unigenes with an average length of 906 base pairs and a N50 of 2318 base pairs were generated. A total of 66,041 unigenes were generated with an average length of 922 base pairs and 2206 base pairs at N50 level for *Anser cygnoides*. The length of distribution of the assembled unigenes for both species is illustrated in Figure 1. The GC content of the assembled unigenes for *Anser anser* and *Anser cygnoides* was less variable between the species, each with an average of 49% and 50% GC, respectively. 

### 3.2. Functional Annotation of Gene Ontology (GO), Eukaryotic Orthologous Groups (KOG), and Kyoto Encyclopedia of Genes and Genomes (KEGG)

**Figure 2:**
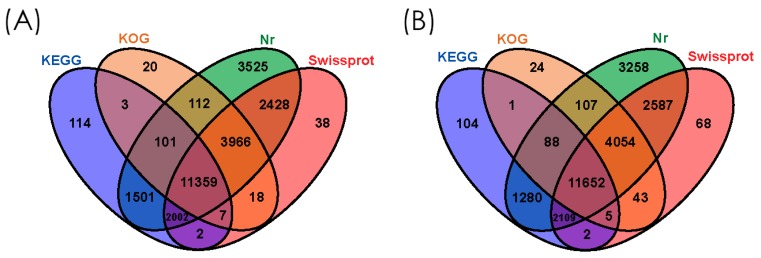
Venn diagrams showing the unigene assembly BLAST results of *Anser anser* (**A**) and *Anser cygnoides* (**B**) transcriptome against four databases. These show the differences and similarities of the number of unigenes annotated in each database within the individual species and between different species.

Annotation of the *Anser anser* and *Anser cygnoides* assembled unigene sequences were performed using BLASTX (E value ≤ 1 × 10^−5^) searched against the NCBI non-redundant protein (Nr) database, the Swiss-Prot protein database, Eukaryotic Orthologous Groups (KOG), and Kyoto Encyclopedia of Genes and Genomes (KEGG) (Figure 2). A total of 77,134 for *Anser anser* and 66,041 for *Anser cygnoides* unigenes were annotated. From the above-annotated sequences for both species, most of the genes had insignificant (without annotation genes) BLAST hits to the four databases; 51,938 for *Anser anser* and 40,659 for *Anser cygnoides*. Generally, the Nr database annotated more unigenes for both species with 24,994 (32.40%), followed by the Swiss-Prot database with 19,820 (25.70%), 15,586 (20.21%) in the KOG database, and 15,089 (19.56%) in KEGG for *Anser anser* whereas *Anser cygnoides* annotated 25,135 (38.06%) in the Nr database, 20,520 (31.07%) in the Swiss-Prot database, 15,974 (24.19%) in the KOG database, and 15,241 (23.08%) in the KEGG database. More noticeably, all of the employed databases had a higher E-value distribution hits (strong homology) E-value from 1 × 10^−150^ in both species.

**Figure 3:**
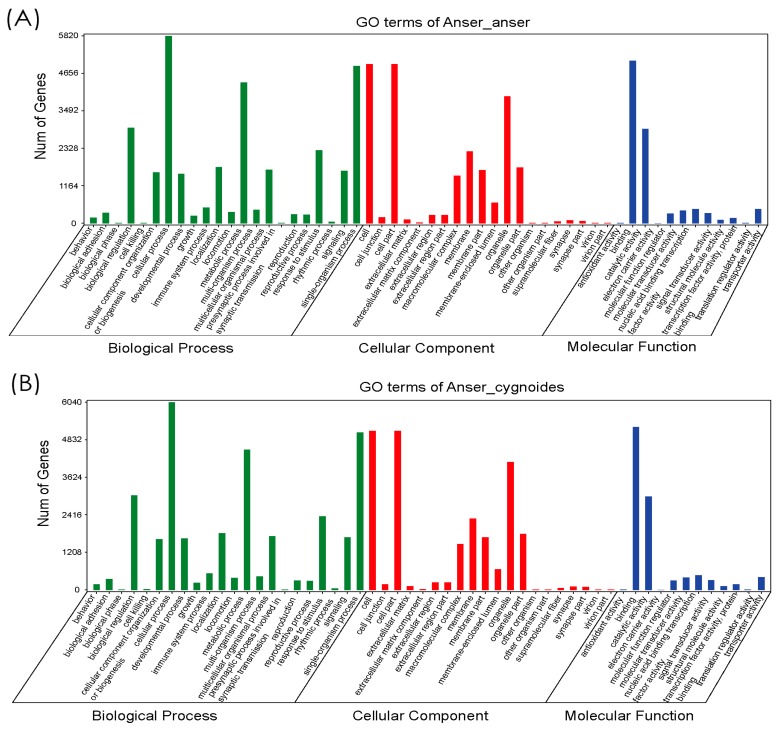
Comparison of the GO functional annotations of unigenes between the *Anser anser* (**A**) and *Anser cygnoides* (**B**) species into three major categories: biological process, cellular component, and molecular function.

The annotated sequences of 19,926 unigenes from *Anser anser* and 20,515 from *Anser cygnoides* were assigned to Gene Ontology (GO) terms, which were classified into three GO categories known as biological process, cellular component, and molecular functions (Figure 3A,B). For both species, the gene distribution in each of the GO categories was comparatively similar. In the biological processes category, cellular process (5814, 6035) was predominant, followed by single-organism process (4886, 5067), metabolic process (4373, 4514), and biological regulation (2962, 3045). In the cellular component category, cell and cell part showed a predominant number of genes in both species (4945, 5114; 4945, 5114), followed by organelle (3945, 4116), and membrane (2235, 2298). In molecular function, binding (5048, 5244) and catalytic activity (2930, 3004) were prominently observed.

**Figure 4:**
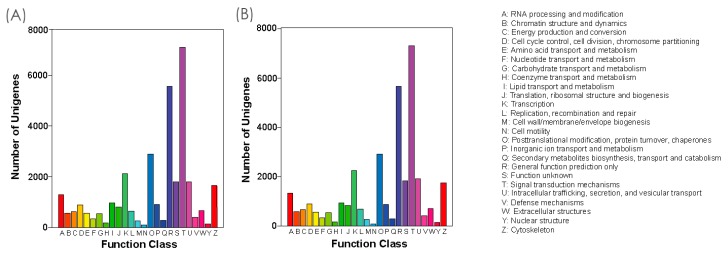
Transcriptomes of Eukaryotic Orthologous Groups (KOG) classifications for *Anser anser* (**A**) and *Anser cygnoides* (**B**). To identify and classify possible functions, all unigenes were aligned to the KOG database.

Orthologous unigene functional prediction and classification of *Anser anser* and *Anser cygnoides* were performed using the KOG database. The unigenes were assembled to 25 KOG categories (Figure 4A,B), from which both species had a similar trend of functional classes. We found that the cluster for “signal transduction mechanisms” represented the largest group among all of the groups (7121 (21.58%) for *Anser anser* unigenes and 7315 (21.63%) for *Anser cygnoides* unigenes, followed by “general function prediction only” (5575 (16.89%); 5659 (16.73%)), “posttranslational modification, protein turnover, chaperones” (2886 (8.74%); 2912 (8.61%)), and “transcription” (2116 (6.41%); 2243 (6.63%)). Additional detailed information of all clusters is provided in Table S1. 

**Figure 5:**
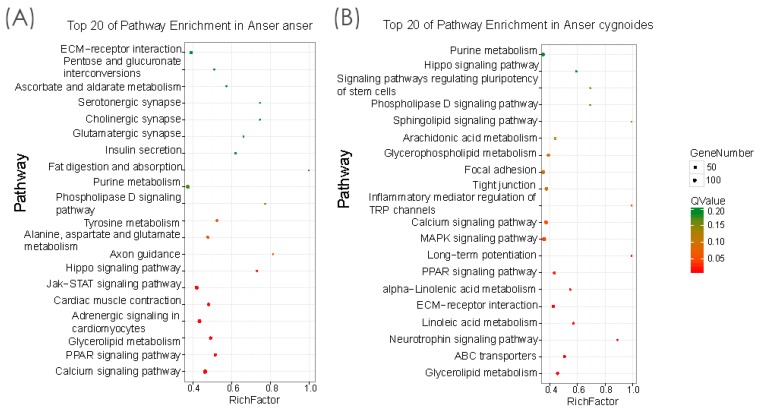
Top 20 enriched KEGG pathways for *Anser anser* (**A**) and *Anser cygnoides* (**B**). The *y*-axis represents the name of the pathway, and the *x*-axis represents the rich factor and the ratio of the number of DEGs and the number of all genes in the pathway is represented by the rich factor. The Qvalue indicates the significant enrichment of the DEGs and pathways.

Furthermore, to investigate the potential difference in signaling pathways between *Anser anser* and *Anser cygnoides,* the annotated unigenes were mapped to the KEGG database. In this study, we found 218 pathways were mapped for *Anser anser* and 216 pathways for *Anser cygnoides,* showing variation in the contribution for each pathway in a species-specific manner (Tables S2 and S3). Amongst the top 20 enriched pathways, ten pathways in *Anser anser* were significantly enriched at levels (Qvalue ≤ 0.05) namely “calcium signaling pathway (ko04020)”, “PPAR signaling pathway (ko03320)”, “glycerolipid metabolism (ko00561)”, “adrenergic signaling in cardiomyocytes (ko04261)”, “cardiac muscle contraction (ko04260)”, “Jak-STAT signaling pathway (ko04630)”, “hippo signaling pathway (ko04390)”, “axon guidance (ko04360)”, “alanine, aspartate and glutamate metabolism (ko00250)”, and “tyrosine metabolism (ko00350)”, respectively (Figure 5A). In *Anser cygnoides* amongst 20 top enriched pathways, 11 pathways were significantly enriched at the level (Qvalue ≤ 0.05) including “glycerolipid metabolism (ko00561)”, “ABC transporters (ko02010)”, “neurotrophin signaling pathway (ko04722)”, “linoleic acid metabolism (ko00591)”, “ECM-receptor interaction (ko04512)”, “alpha-linolenic acid metabolism (ko00592)”, “PPAR signaling pathway (ko03320)”, “long-term potentiation (ko04720)”, “ MAPK signaling pathway (ko04010)”, calcium signaling pathway (ko04020)”, and “inflammatory mediator regulation of TRP channels (ko04750)”, successively (Figure 5B).

### 3.3. Gene Homology Analysis

**Figure 6:**
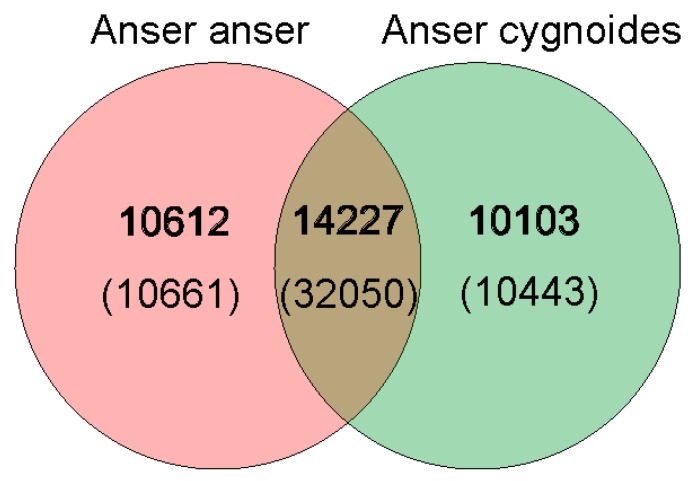
Gene families and genes obtained from the transcriptomes of *Anser anser* and *Anser cygnoides*. Gene families and genes were identified from the two transcriptomes using BLASTp and an OrthoMCL, with the total number of gene families and genes belonging to each species shown in the figure with 14,227 gene families and 32,050 genes conserved between the two species’ transcriptomes.

BLASTp was used in conjunction with OrthoMCL to find homogenes of homology between species. Ideally, the sequences were aligned in pairs and in the BLAST alignment, the pair of genes with an E value of less than 1 × 10^−7^ was considered to be a homologous gene between species. OrthoMCL was used to classify genes that were homologous to each other into the same family. We found a total of 34,942 gene families based on sequence alignment homology and 53,154 genes, of which 24,839 families and 42,711 genes belonged to *Anser anser*, and 24,330 gene families with 42,493 genes belonged to *Anser cygnoides*. In the comparative transcriptome of the two species, we identified 14,227 gene families and 32,050 genes conserved between *Anser anser* and *Anser cygnoides*. We further identified 10,612 gene families and 10,661 genes for *Anser anser*, and 10,103 gene families and 10,443 genes for *Anser cygnoides* that were differentially expressed (Figure 6). We identified the specific gene families of each species to annotate their functions and analyze their KEGG and GO enrichment. The annotations of specific genes in *Anser anser* and *Anser cygnoides* are listed in Tables S4 and S5, respectively.

### 3.4. Differentially Expressed Genes (DEGs) Analysis

In order to identify the differentially expressed genes, the unigenes from the significantly enriched pathway in each species were randomly selected. We identified pathways commonly enriched in GO terms for hair follicle and skin development (Table 2). We further identified various genes, which contributed significantly to geese embryonic skin feather follicle development between the two species (Table 3). 

### 3.5. Validation of RNA-Seq by RT-qPCR

**Figure 7:**
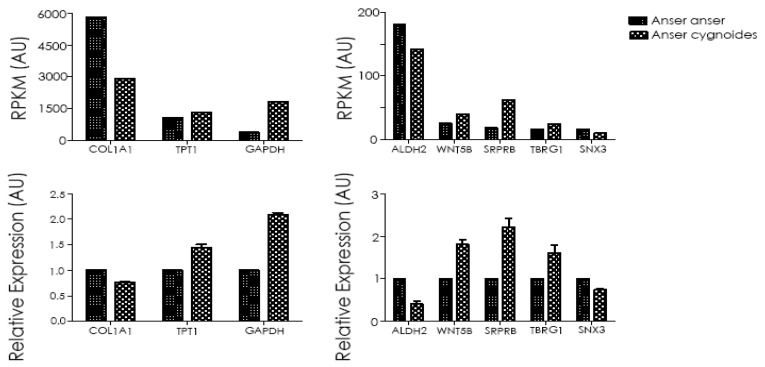
RNA-Seq data reliability check by RT-qPCR. Panel A indicates the RNA-Seq expression level (RPKM) defined as the False Discovery Rate (FDR) < 0.05 and |log2 Fold Change| > 1 and panel B represents the mRNA expression level calculated as the 2^−ΔΔCt^ method of the selected genes.

The reliability of the RNA-Seq data was validated by RT-qPCR through the random selection of eight DEGs: *COL1A1*, *TPT1*, *GAPDH*, *ALDH2*, *WNT5B*, *SRPRB*, *TBRG1*, and *SNX3*. For comparison, in the *Anser anser* species, the DEGs *COL1A1*, *ALDH2*, and *SNX3* were upregulated, whereas *TPP1, GAPDH*, *WNT5B*, *SRPRB*, and *TBRG1* were downregulated. The expression patterns of RT-qPCR data showed a consistent trend with the RNA-Seq results and hence validated the authenticity of the transcriptome (Figure 7).

## 4. Discussion

**Figure 8:**
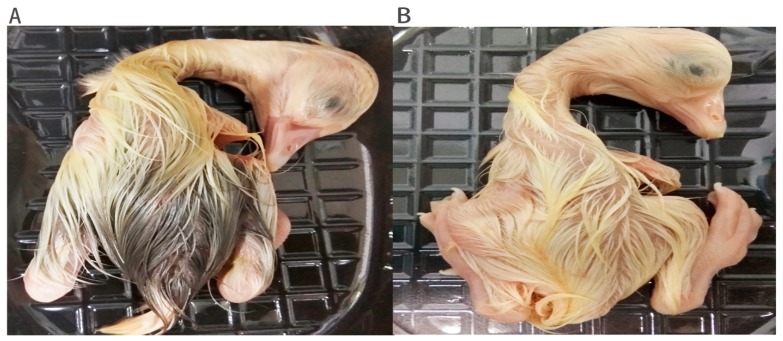
The *Anser anser* and *Anser cygnoides* fluffy embryonic downy pigmentation. (**A**) *Anser anser*; (**B**) *Anser cygnoides*.

Regional variations in feather color on an individual body are referred to as pigment patterning [21]. Feather distribution and pigmentation patterns can be distinctive at different stages of a bird’s life span [22]. Poultry melanin is produced by melanocytes within feathers (from a horizontal ring at the proximal base of the feather follicle) and moves to the barb ridges to produce pigments during feather development [23]. Furthermore, different bird species display diverse genetic mechanisms that entail specific spatio-temporal regulation, which leads to distinctive pigmentation during development [24]. The authors in [25] reported the appearance of different feather pigmentation patterns in certain regions of quail embryo at different stages of development. The variation in feather pigmentation pattern between the two experimental geese species during embryonic growth is illustrated in Figure 8.

In the past few years, there have been numerous studies on the complex signaling pathway mechanisms regulating skin and hair/feather follicle growth/development in mammals. However, the significant difference in the molecules and signaling transduction pathways controlling embryonic skin feather follicles between the closely related poultry species is not fully understood. The difference between avian feather follicle development might be associated with complex hierarchical branching patterns of the ectodermal organ that control endothermy, communication, and flight [26]. To further elucidate the differentially expressed genes between the two geese species, KEGG signaling pathway analysis was performed. The major commonly shared signaling pathways in the top 20 enriched pathways between the two species’ embryonic skin feather follicles included the calcium signaling pathway, PPAR signaling, glycerolipid metabolism, the hippo signaling pathway, the phospholipase D signaling pathway, purine metabolism, and ECM–receptor interaction. Among others, some of these signaling pathways have been predicted in previous studies and are involved in the regulation of human skin development, specifically the differentiation of the epidermis [27], in chicken feather/scale development [28], hair follicle morphogenesis in cashmere goat (*Capra hircus*) [29], and mouse skin development [30]. Moreover, it is worth noticing that there were some signaling pathways present in a species-specific-manner at a significant level (Qvalue ≤ 0.05), adrenergic signaling in cardiomyocytes, cardiac muscle contraction, Jak-STAT signaling pathway, and axon guidance in *Anser anser*. For *Anser cygnoides*, they included ABC transporters, neurotrophin signaling, linoleic acid metabolism, Alpha-Linoleic acid metabolism, and long-term potentiation. These differences may be attributed to the contribution of the genes involved in these pathways to the phenotypic expression differences in feather color, quality, and quantity between the species. In this study, we found that the calcium signaling pathway and glycerolipid metabolism were the major pathways involved in the embryonic skin development of the two species. Calcium deficiency may be associated with poor quality feathers that break easily and have a dull, ragged-looking appearance [31]. Additionally, [32] found that calcium enrichment increased the number of black feather growth (feather melanization) and hence the size of the black plumage patch. Glycerolipids form the major components of sebum (surface lipid), contributing to bat communication, innate immune system, compound adsorption, pheromone release, organization of the stratum corneum, ultraviolet protection, hydration, and water repellency [33]. Therefore, we suggest that the genes belonging to these pathways could be target genes, advocating further understanding of the molecular mechanisms regulating feather follicles development between the two species.

The family of Wnt proteins signaling secretes highly conserved molecules that play a vital role during embryogenesis including cell differentiation, cell proliferation, and cell degeneration determination, thus regulating growth in many biological events [34]. In this study, we found that the *Wnt6* gene was differentially expressed between the two species in the regulation of biological processes. The study in [35] found that *Wnt6* was also differentially expressed during embryonic skin feather formation. Neurotrophins and their receptors’ proteins family do not only participate in neuronal cell survival and differentiation, but also contribute to certain biological activities on nonneuronal tissues such as skin [36]. A previous report by [37] indicated that *NGFR* was expressed in a multiple number of cell types derived from the inner endoderm mesoderm and surface ectoderm. Our study revealed that *NGFR* was expressed differentially in the feather follicle development of the geese *Anser anser* and *Anser cygnoides*. The fibroblast growth factors (FGFs) and their receptors are the main regulators of organogenesis during early stages of embryonic development, tissue repair, and regeneration in adults [38]. The FGF family is divided into seven subfamilies consisting of 22 polypeptides that control metabolic activity, survival, migration, proliferation, neural functions, and differentiation in various cells [39]. The authors in [40] found that *FGF16* was expressed in brown adipose tissue during embryonic development in rats. Our results also showed that *FGF16* was differentially expressed for the developmental process in the embryonic skin feather follicles in geese. Bone morphogenetic proteins (BMPs) are the largest members of secreted signaling molecules belonging to the transforming growth-beta (TGF-b) and their mutual action with distinct receptors impose several important biological processes [41]. BMPs regulate cell proliferation, differentiation, and apoptosis in different body cell/tissue types including developing epidermis, hair follicle growth, and melanogenesis in embryonic and postnatal skin [42]. The study by [43] indicated that *BMP7* was expressed throughout the epidermis before placode formation and was consequently constrained to the primordium in both the dermis and epidermis. We found that *BMP7* was upregulated in *Anser anser,* suggesting that BMP might have an indispensable contribution to the epithelium development when compared to the *Anser cygnoides* species. Histone deacetylase 2 (HDAC2) is a member of the class I histone deacetylases, which catalyzes the deacetylation of histones and acts as a promoter and a coding region of transcribed genes and regulates chromatin structure and transcription [44]. *HDAC2* was expressed in the outer root sheath, hair matrix, and epidermal layer of the skin, and the epidermal suppression of *HDAC2* activity indirectly impacts pigmentation, resulting in abnormal ectodermal organ morphogenesis, interrupted hair follicle homeostasis, and regeneration [45]. In [46], it was revealed that *HDAC2* expression varied during epidermal cell development in mice. In this study, we found that *HDAC2* was involved in the embryonic skin development in both species, but was downregulated in *Anser cygnoides*. This might be associated with the minimal contribution of *HDAC2* in skin feather follicle development in *Anser cygnoides*. Insulin-like growth factor 1 receptor (IGF-1R) is a member of an insulin like growth factor receptor (IGFR) family of growth hormones [47]. *IGF-1R* mediates the action of insulin growth factor 1 (IGF-1), which is locally produced by mesenchyme-derived cells. Subsequent activation of heterotetrameric *IGF-1R* leads to its auto-phosphorylation and function as a base for multiple signaling transduction pathways including the activation of mitogen-activated protein kinase (MAPKs) and phosphatidylinositol 3-kinase (PI3Ks) [48]. Immunity-related cells (some thymocytes, natural killer cells, some T lymphocytes, monocytes, and most B cells) expressed *IGR-1R* depending on the stimulation conditions [49]. These study findings revealed that *IGF-1R* participated in the morphogenesis of an epithelium upregulated in *Anser anser* and downregulated in *Anser cygnoides,* which implies that *IGF-1R* might play an imperative role in controlling and regulating skin formation and feather development. During embryogenesis, the formation of blood vessels occurs through vasculogenesis, thus the primitive vascular network forms from the differentiation of undifferentiated angioblasts into endothelial cells [50]. Vascular endothelial growth factor-C (VEGF-C) is a known member of the vascular endothelial growth factor (VEGF) family that has different functions such as endothelia regulatory factors [51]. The authors [52] found that hair growth in rabbits was significantly accelerated by *VEGF-C* through the activation of cells controlling the regulation of the hair growth cycle through growth factors. Our results showed that *VEGF-C* was differentially expressed between two species contributing to biological regulation in the focal adhesion signaling pathway, thus *VEGF-C* may play an important role in geese angiogenesis. Platelet-derived growth factor (PDGF) is a potent mitogen consisting of three different polypeptides (PDGF-A, PDGF-B, and PDGF-C) produced in numerous types of cells such as endothelial and keratinocyte cells and is essential for proliferation, differentiation, and growth [53]. *PDGFB* was observed in the keratinocytes cells of the hair follicles and not detected in the cultured dermal papillae cells in humans [54]. Our research found that *PDGFB* was predominantly expressed in the *Anser anser* species and comparatively less in *Anser cygnoides* embryonic skin. This suggests that *PDGFB* might have a substantial role in the epithelium development of *Anser anser* to enhance cell proliferation and differentiation. Taken together, the selected differentially expressed genes were more pronounced in the *Anser anser* geese species, suggesting that these genes might have potential in regulating feather formation and hence improving the quality of downy feathers.

Ultimately, the RNA-Seq results were validated by RT-qPCR to access the relative expression levels of the eight randomly selected DEGs (*COL1A1*, *TPT1*, *GAPDH*, *ALDH2*, *WNT5B*, *SRPRB*, *TBRG1*, and *SNX3*), which showed a consistent trend of expression, therefore authenticating our results’ reliability. Skin connective tissue is comprised mainly of collagens type I and III, representing about 80% of the dermis and confer stress resistance mechanisms providing protection from skin tearing and deformation [55]. *COL1A1* is a type 1 collagen that regulates cell proliferation, migration, and control gene expression [56]. The present reports on *COL1A1* have focused mainly on bone diseases, tumor tissues, osteoporosis, and esteogenesis [57,58]. In our present study, *COL1A1* was upregulated for *Anser anser* in both RNA-Seq and RT-qPCR, suggesting that *COL1A1* may have a key functional role in geese embryonic skin feather follicle development. However, there is a scarcity of information on the molecular mechanism of *COL1A1* involvement in controlling geese feather formation and development.

## 5. Conclusions

In conclusion, by using the RNA-Seq technique, we were able to establish transcriptome profile characterization of the embryonic skin feather follicles of the two geese species with different potentials for the production performance quality of downy feathers. A total of 170,246,914 raw reads for *Anser anser* and 147,754,320 raw reads for *Anser cygnoides* were obtained. De novo assembly by Trinity generated 165,564,278 and 144,595,262 clean reads for *Anser anser* and *Anser cygnoides*, respectively, which were further assembled into 77,134 unigenes with an average length of 906 base pairs in *Anser anser* and 66,041 unigenes with an average length of 922 base pairs in *Anser cygnoides.* This study further identified differentially expressed genes such as *IGF1R*, *VEGFC*, *PDGFB*, and *KCNMA1*, and significantly enriched signal transduction pathways, particularly the calcium signaling pathway and glycerolipid metabolism, through the help of bioinformatics software to provide a deep insight into the genomic variations between the two related species, which might be responsible for the quality of feathers. The identification of genes and involved signaling transduction cascades can be targets for further study in promoting high-grade downy feathers and assist in skin-related studies in other animals.

## Figures and Tables

**Table 1:** Transcriptome sequencing summary of *Anser anser* and *Anser cygnoides*.

	*Anser anser*	*Anser cygnoides*
Total number of raw reads	170,246,914	147,754,320
Total number of clean reads	165,564,278	144,595,262
Total length of clean reads (bp)	24,315,965,489	21,274,765,140
Q20 percentage after filter (%)	98.31	98.46
Total number of unigenes	77,134	66,041
GC percentage of unigenes (%)	49.3170	49.8801
N50 length of unigenes (bp)	2318	2206
Max length of unigenes (bp)	24,412	22,078
Min length of unigenes (bp)	201	201

**Table 2:** Statistically common enriched GO terms (GO: 0001942//hair follicle development; GO: 0043588//skin development) between *Anser anser* and *Anser cygnoides*.

Pathways	DEGs in *Anser anser* (2200)	All genes in *Anser anser* (6850)	DEGs in *Anser cygnoides* (2100)	All genes in *Anser cygnoides* (6777)	Pathway ID
Cytokine-cytokine receptor interaction	71 (3.23%)	230 (3.36%)	49 (2.33%)	210 (3.1%)	ko04060
Adherens junction	26 (1.18%)	98 (1.43%)	48 (2.29%)	120 (1.77%)	ko04520
Cell cycle	32 (1.45%)	170 (2.48%)	55 (2.62%)	190 (2.8%)	ko04110
TGF-beta signaling pathway	24 (1.09%)	108 (1.58%)	17 (0.81%)	95 (1.4%)	ko04350
Wnt signaling pathway	52 (2.36%)	182 (2.66%)	50 (2.38%)	192 (2.83%)	ko04310
Endocytosis	125 (5.68%)	388 (5.66%)	120 (5.71%)	376 (5.55%)	ko04144
Metabolic pathways	569 (25.86%)	1688 (24.64%)	513 (24.43%)	1623 (23.95%)	ko01100
MAPK signaling pathway	129 (5.86%)	372 (5.43%)	149 (7.1%)	393 (5.8%)	ko04010
Hedgehog signaling pathway	16 (0.73%)	61 (0.89%)	18 (0.86%)	61 (0.9%)	ko04340

**Table 3:** The KEGG pathway and GO function of DEGs associated with embryonic skin feather follicle development.

Symbol	*Anser anser*		*Anser cygnoides*		KEGG Pathway	GO Function
	Gene ID	RPKM	Gene ID	RPKM		
*Wnt6*	Unigene0008642	5.0506	Unigene0034364	3.4138	ko04310//Wnt signaling pathway	GO:0050789//regulation of biological process
*NGFR*	Unigene0002367	24.2656	Unigene0045291	12.6814	ko04060//Cytokine-cytokine receptor interaction	GO:0001942//hair follicle development
*FGF16*	Unigene0016221	2.0958	Unigene0019329	0.8415	ko04010//MAPK signaling pathway	GO:0032502//developmental process
*BMP7*	Unigene0043457	1.8824	Unigene0010378	0.6135	ko04060//Cytokine-cytokine receptor interaction	GO:0060429//epithelium development
*HDAC2*	Unigene0039479	17.7176	Unigene0022448	0.0936	ko04110//Cell cycle	GO:0043588//skin development
*IGF1R*	Unigene0054392	2.1838	Unigene0005210	1.695	ko04144//Endocytosis	GO:0002009//morphogenesis of an epithelium
*VEGFC*	Unigene0019611	3.3092	Unigene0022184	2.561	ko04510//Focal adhesion	GO:0065007//biological regulation
*PDGFB*	Unigene0053351	8.9492	Unigene0044387	5.1886	ko04010//MAPK signaling pathway	GO:0060429//epithelium development
*KCNMA1*	Unigene0054701	0.4124	Unigene0042712	0.6262	ko04270//Vasc-ular smooth muscle contraction	GO:0008544//epidermis development

## Supplementary Materials

The following are available online at https://www.mdpi.com/2073-4425/10/5/351/s1, Table S1: Detailed information of the 25 KOG categories, Table S2: Pathway Enrichment in the *Anser anser*, Table S3: Pathway Enrichment in the *Anser cygnoides*, Table S4: Annotations of specific genes in the *Anser anser*, Table S5: Annotations of specific genes in the *Anser cygnoides*.
